# Increased red cell distribution width predicts severity of drug-induced liver injury: a retrospective study

**DOI:** 10.1038/s41598-020-80116-4

**Published:** 2021-01-12

**Authors:** Xu Li, Hongqin Xu, Pujun Gao

**Affiliations:** 1 Department of Hepatology, The First Hospital of Jilin University, Jilin University, No. 71 Xinmin Street, Changchun, 130021 China; 2 Key Laboratory of Organ Regeneration and Transplantation of Ministry of Education, The First Hospital of Jilin University, Changchun, 130061 People’s Republic of China; 3 Jilin Province Key Laboratory of Infectious Disease, Laboratory of Molecular Virology, Changchun, 130021 China

**Keywords:** Diseases, Liver fibrosis

## Abstract

We used a retrospective study design to evaluated the predictive value of red cell distribution width (RDW) for drug-induced liver injury (DILI) severity in Chinese patients with liver biopsy to assist with early DILI management. We included 164 DILI patients with complete laboratory information and medical history. We compared outcomes of 36 patients with severe DILI with outcomes of a control group of 128 patients with mild-to-moderate DILI. Multivariate analyses of risk factors for severe liver injury in Chinese patients with DILI revealed an estimated adjusted odds ratio (AOR) (95% CI) of 4.938 (1.088–22.412) in patients with drinking. Risk for serious liver injury was also increased significantly in patients with dyslipidemia [AOR (95% CI) 3.926 (1.282–12.026)], higher serum total bile acid (TBA) levels [AOR (95% CI) 1.014 (1.009–1.020)] and higher RDW [AOR (95% CI) 1.582 (1.261–1.986)]. The result for area under the curve of 0.905 for TBA levels indicated this variable had high diagnostic performance for predicting DILI severity. Based on an area under the curve value of 0.855, RDW also had superior diagnostic performance in prediction of DILI severity. This performance was not significantly different compared with TBA and was superior compared with other variables, which had area under values ranging from poor to failure (0.527–0.714).The risk for severe DILI was associated with drinking, dyslipidemia, higher TBA levels and RDW values. This study found that RDW and TBA levels were predictors of DILI severity in Chinese patients.

## Introduction

Although limited data exist on the incidence among the general population of drug-induced liver injury (DILI), it is an important public health problem because many medications are likely involved in the occurrence of liver injury^1,2^. Because DILI is a difficult disease to diagnose, the actual values for incidence are likely greater than reported. Diverse individual characteristics that contribute to specific host-drug interactions result in a variety of clinical presentations that complicate the diagnosis of DILI^3,4^. Some degree of repair to liver damage typically occurs when the drug or drugs are stopped. However, in a small percentage of patients, DILI can persist and progress after drug removal.

Variability in red blood cell (RBC) size can be measured using red blood cell distribution width (RDW). RDW measurement is typically included in complete blood cell counts^5–7^. Study results indicate that RDW can be used as an independent predictor of the prognosis of diseases including renal diseases, cardiovascular diseases, pulmonary hypertension, lung cancer, and sepsis^8–14^. Significant associations have been found between RDW and chronic liver disease severity (e.g., nonalcoholic fatty liver disease, alcoholic cirrhosis, and chronic hepatitis B (CHB))^15–17^. Elevated RDW levels have been found in patients with autoimmune liver disease such as primary biliary cholangitis and autoimmune hepatitis^18,19^. DILI progression and severity is associated with inflammation and autoimmune reaction^20^. However, the association between RDW values and DILI severity is mostly unknown.

The objective of this study was to analyze risk factors for DILI severity in a Chinese population. We evaluated and compared the performance of noninvasive serum sample-associated markers (i.e., total bile acid (TBA) levels, RDW, RDW-to-platelet count (PLT) ratio (RPR), RDW-to-lymphocyte ratio (RLR), and aspartate aminotransferase-to-alanine aminotransferase ratio (AAR) when used to diagnosis disease severity in 164 Chinese patients with DILI.

## Results

### Demographic and clinical characteristics

Patient demographic and clinical characteristics are presented in Tables 1 and 2. The study population consisted of 24.4% men. The median age was 50.0 (42.0, 56.0) years. The values for prevalence of hypertension and dyslipidemia were 15.9% (26/164) and 36% (59/164), respectively. The prevalence of diabetes mellitus and a history of hypersensitivity were 8.5% (14/164) and 17.7% (29/164), respectively.

**Table 1:** Baseline characteristics of the study population.

Variable	Total DILI (N = 164)	Normal range
Male, N(%)	40 (24.4)	
Age (years)	50.00 (42.00, 56.00)	
Smoking, N(%)	24 (14.6)	
Drinking, N(%)	20 (12.2)	
Hypersensitivity history, N(%)	29 (17.7)	
Hypertension, N(%)	26 (15.9)	
Dyslipidemia, N(%)	59 (36.0)	
Diabetes, N(%)	14 (8.5)	
WBC count (10^9^/L)	4.98 (3.94, 6.47)	3.50–9.50
Neutrophil (10^9^/L)	2.74 (1.96, 3.59)	1.80–6.30
Lymphocyte (10^9^/L)	1.69 (1.24, 2.19)	1.10–3.20
Hemoglobin (g/L)	128.00 (120.00, 140.00)	115–150
Platelet (10^9^/L)	201.00 (164.00, 253.00)	125–350
RDW (%)	14.30 (13.10, 15.83)	11.0–14.0
MCV (fL)	89.90 (86.40, 93.30)	82–100
AST (IU/L)	117.80 (46.55,230.60)	13.0–35.0
ALT (IU/L)	164.35 (55.03,330.85)	7.0–40.0
TBIL (μmol/L)	29.40 (13.70,122.13)	0.0–6.8
ALP (IU/L)	121.90 (96.80,189.78)	35.0–100.0
GGT (IU/L)	147.65(77.40,275.53)	7.0–45.0
Globulin (g/L)	28.40 (24.60,31.85)	20.0–40.0
TBA (μmol/L)	25.60 (8.20,136.45)	0–10
PT (s)	11.45(10.80,12.40)	9.0–13.0
INR	0.99(0.93, 1.06)	0.8–1.2

Continuous variables are expressed as median (25th, 75th percentiles) values. Categorical variables were displayed as numbers and percentages.

*WBC* white blood cell, *RDW* red blood cell distribution width, *MCV* mean corpuscular volume, *AST* aspartate aminotransferase, *ALT* alanine aminotransferase, *TBIL* total bilirubin, *ALP* alkaline phosphatase, *GGT* gamma-glutamyltransferase, *TBA* total bile acid, *PT* prothrombin time, *INR* international normalized ratio.

**Table 2:** Clinical characteristics of 164 patients with drug-induced liver injury.

Symptoms	No of cases (%)	Laboratory results	No of cases (%)
Fatigue	101 (61.6)	AST elevation	140 (85.4)
Abdominal distension	53 (32.3)	ALT elevation	141 (86.0)
Jaundice	69 (42.1)	GGT elevation	145 (88.4)
Nausea	24 (14.6)	ALP elevation	118 (72.0)
Pruritus	1 (0.61)	TbiL elevation	101 (61.6)
None	20 (12.2)	TBA elevation	114 (69.5)

*AST* aspartate aminotransferase, *ALT* alanine aminotransferase, *GGT* gamma-glutamyl transferase, *ALP* alkaline phosphatase, *TbiL* total bilirubin, *TBA* alkaline phosphatase.

Clinical characteristics of patients with DILI are shown in Table 2. The most prevalent clinical manifestations were fatigue (61.6%) and jaundice (42.1%). Abdominal distension and nausea accounted for 32.3% and 14.6%, respectively. A total of 12.2% of the patients had no symptoms or clinical signs.

### Therapeutic classes and uses of drugs

The therapeutic classes of drugs used by participants in the DILI group are listed in Table 3. Of note, 79 DILI patients (48.2%) used Chinese herbal medicines, 56 (34.1%) used Western medicines, and 29 (17.7%) used a combination of the two. These drugs are suspected to be the cause of liver injury in DILI patients. To further evaluate the indication of the herbal drugs, we subdivided the 79 patients who used Chinese herbal medications into causal categories (Table 4). Dietary supplements, anti-inflammatory drugs, Cardiovascular drugs, Osteoarthropathy drugs, and digestive system drugs were the top five types of causal herbal drugs.

**Table 3:** Therapeutic classes of drugs that caused liver injury in 164 Chinese patients.

	No. of cases	Percentage
Chinese herbal medicines	79	48.2
Western medicines	56	34.1
Both	29	17.7

**Table 4:** Indications of drugs that caused liver injury in 79 Chinese DILI patients, induced by herbal medications.

Cause	No of cases
Dietary supplements	30
Anti-inflammatory drugs	12
Cardiovascular drugs	10
Osteoarthropathy drugs	7
Digestive system drugs	7
Obstetric/gynecological drugs	4
Rheumatism drugs	3
Nervous/mental system drug	3
Others	9

*DILI* drug-induced liver injury.

### Univariate and multivariate analyses

We evaluated risk factors for severity of DILI in the 164 patients who had the condition (Table 5). The results for the univariate analysis indicated that drinking, dyslipidemia, TBA level, RDW level, RLR level, and AAR level were significantly different between patients with severe (≥ level 3) and mild-to-moderate (levels 0–2) disease. Sex, age, smoking, drinking, history of hypersensitivity, diabetes mellitus, history of liver disease, dyslipidemia, TBA, RDW, RPR, RLR, and AAR were included in the multivariate analysis. The AOR for patients with dyslipidemia was 3.926 (95% CI 1.282–12.026; *P* = 0.017), compared with patients with normal plasma lipid values. The AOR for patients who drank was 4.938 (95% CI 1.088–22.412; *P* = 0.039), compared with patients who did not drink. Compared with patients with lower TBA levels, patients with higher TBA levels had a higher risk for development of severe DILI (1.014 [95% CI 1.009–1.020], *P* < 0.001). Patients with higher RDW values had a higher risk for development of severe DILI (1.582 [95% CI 1.261–1.986], *P* < 0.001). We found no significant associations between levels of RPR, RLR, AAR, and DILI severity.

**Table 5:** Univariate and multivariate analyses of variables associated with severe drug-induced liver injury.

Variables	Level 0–2 (N = 128)	Level ≥ 3 (N = 36)	*P*^*#*^	AOR (95% CI)*	P**
**Sex**			0.157	–	–
Female, N(%)	100 (78.1)	24 (66.7)			
Male, N(%)	28 (21.9)	12 (33.3)			
**Age**			0.353	–	–
< 60	108 (84.4)	28 (77.8)			
≥ 60	20 (15.6)	8 (22.2)			
**Smoking**			0.355	–	–
No, N(%)	111 (86.7)	29 (80.6)			
Yes, N(%)	17 (13.3)	7 (19.4)			
**Drinking**			0.017	4.938 (1.088–22.412)	0.039
No, N(%)	117 (91.4)	27 (75.0)			
Yes, N(%)	11 (8.6)	9 (25.0)			
**Hypersensitivity history**			0.096	–	–
No, N(%)	102 (79.7)	33 (91.7)			
Yes, N(%)	26 (20.3)	3 (8.3)			
**Diabetes**			0.737	–	–
No, N(%)	116 (90.6)	34 (94.4)			
Yes, N(%)	12 (9.4)	2 (5.6)			
**History of liver disease**			0.524	–	–
No, N(%)	99 (77.3)	26 (72.2)			
Yes, N(%)	29 (22.7)	10 (27.8)			
**Dyslipidemia**			0.006	3.926 (1.282–12.026)	0.017
No, N(%)	89 (69.5)	16 (44.4)			
Yes, N(%)	39 (30.5)	20 (55.6)			
**INR**			0.180		
< 1.0	73 (57.0)	16 (44.4)			
≧ 1.0	55 (43.0)	20 (55.6)			
TBA	14.75 (7.40, 56.95)	185.60 (132.75, 271.85)	< 0.001	1.014 (1.009–1.020)	< 0.001
RDW	13.75 (12.80, 14.80)	16.65 (14.95, 19.70)	< 0.001	1.582 (1.261–1.986)	< 0.001
RPR	0.07 (0.05, 0.09)	0.07 (0.06, 0.09)	0.617		
RLR	7.61 (6.10, 10.72)	10.68 (8.44, 17.97)	< 0.001		
AAR	0.71 (0.50, 0.96)	0.91 (0.69, 1.15)	0.015		

Continuous variables are expressed as median (25th, 75th percentiles). Categorical variables were displayed as numbers and percentages.

*INR* international normalized ratio, *RLR* red blood cell distribution width to lymphocyte ratio, *RPR* red blood cell distribution width to platelet ratio, *AAR* aspartate aminotransferase to alanine aminotransferase ratio.

^#^*P* value for univariate analysis.

*Adjusted for sex, age, smoking, drinking, allergic history, DM, hyperlipemia, history of liver disease, TBA, RDW, RPR, RLR, and AAR.

**P value for multivariate analysis.

### Diagnostic performance and thresholds of serum models for severity in DILI patients

**Figure 1:**
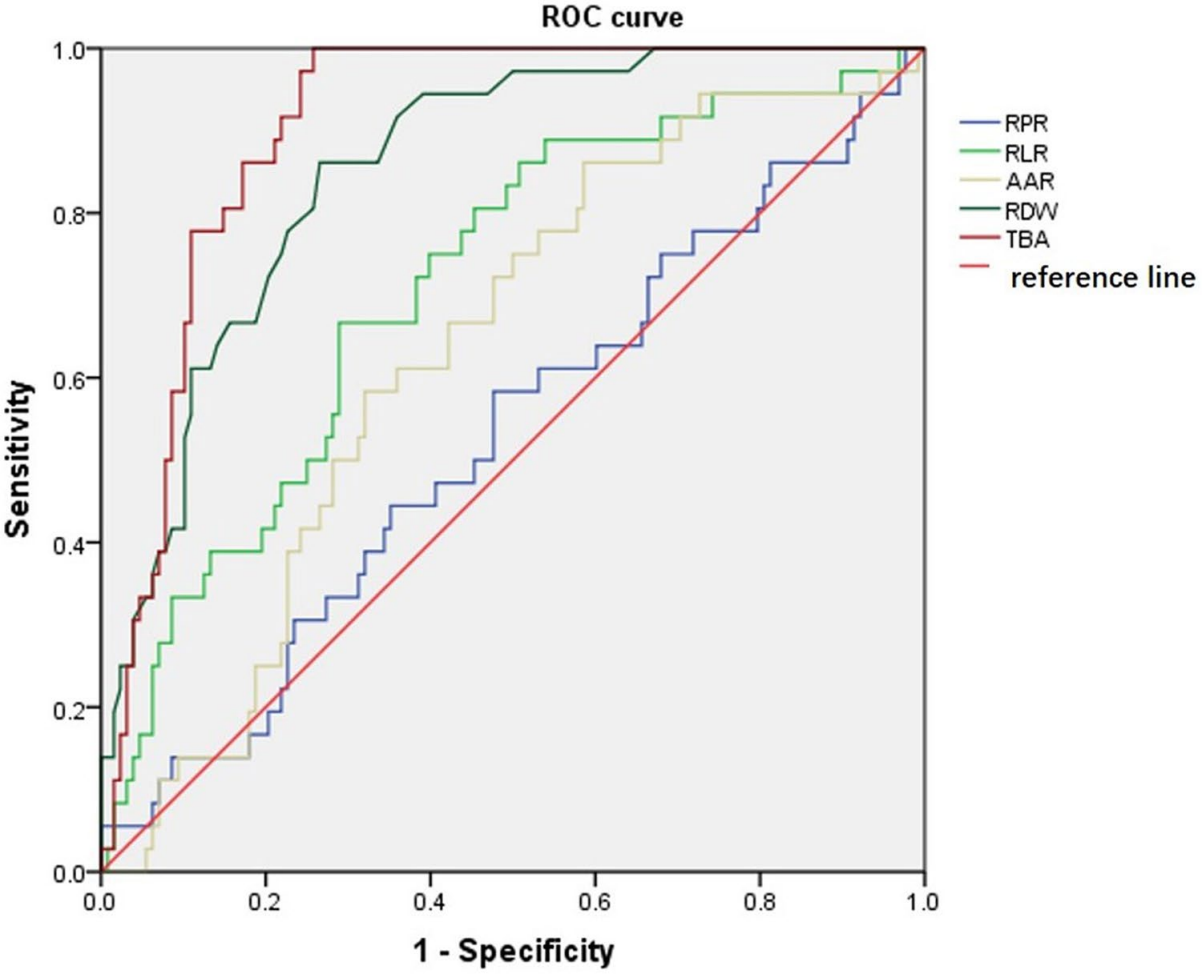
Receiver operating characteristic (ROC) curves of red blood cell distribution width (RDW), RDW-to-platelet ratio (RPR), RDW-to-lymphocyte ratio (RLR), total bile acid (TBA), and aspartate transaminase-to-alanine aminotransferase ratio (AAR) for diagnosis of severe DILI.

The results for the estimation of AUROC values to predict severe DILI indicated that the performance of TBA (AUROC = 0.905, 95% CI 0.860–0.950) and RDW (AUROC = 0.855, 95% CI 0.793–0.917) were higher than RLR (AUROC = 0.714, 95% CI 0.620–0.807), AAR (AUROC = 0.633, 95% CI 0.537–0.728), and RPR (AUROC = 0.527, 95% CI 0.419–0.635) (Table 6, Fig. 1).

**Table 6:** Diagnostic performance of serum models for severe drug-induced liver injury.

Variables	AUROC	(95% CI)	Cut-off	Se (%)	Sp (%)	Correct classified (%)
RPR	0.527	0.419–0.635	0.073	58.3	52.3	53.0
AAR	0.633	0.537–0.728	0.599	86.1	41.4	51.2
RLR	0.714	0.620–0.807	10.227	66.7	71.1	70.1
RDW	0.855	0.793–0.917	14.650	86.1	73.4	76.2
TBA	0.905	0.860–0.950	45.850	100	74.2	79.3
**Comparison of AUROC**						
TBA and RDW	*P* > 0.05					
RDW and RLR	*P* < 0.05					
RLR and AAR	*P* > 0.05					
AAR and RPR	*P* > 0.05					
RLR and RPR	*P* < 0.05					

The bold values were considered statistically significant (*P* < 0.05). Se, sensitivity; Sp, specificity.

Cut-offs were established by maximizing the sum of sensitivity and specificity.

*AUROC* area under the receiver operating characteristic curve, *95% CI* 95% confidence interval.

Maximizing the sum of sensitivity and specificity, the optimal cut-off for TBA was 45.850 for diagnosis of severe DILI. The results indicated that the sensitivity was 100% and the specificity was 74.2% for diagnosis of severe DILI. The correct classified percentage was 79.3%. The optimal cut-off for RDW was 14.650 for diagnosis of severe DILI. The sensitivity was 86.1% and the specificity was 73.4% for diagnosis of severe DILI. The correct classified percentage was 76.2%.

## Discussion

The most important findings of this study were that the results further supported the hypotheses that there was a positive correlation between TBA levels and DILI severity and that RDW predicted DILI severity. At a cutoff of 14.650, RDW predicted severe DILI with a sensitivity of 86.1% and a specificity of 73.4%. The percent correctly classified could be as high as 76.2% with an AUROC of 0.855. These diagnostic capabilities were similar to TBA and were higher than those for other non-invasive markers.

In hepatic diseases, such as viral hepatitis, fatty liver disease, and liver cirrhosis^21,22^, serum bile acid (BA) levels can increase from 10- to 100-fold^21–25^. Compared with serum bilirubin, BA measurement may be a more sensitive method to assess hepatic function^23^. Changes in serum BAs correlate with the severity of hepatic dysfunction. Horvatits et al.’s results indicated that TBA levels can be used as markers to stratify risk in patients with cirrhosis with respect to new onset of acute decompensation and acute-on-chronic liver failure^21^.

Numerous studies have examined associations between RDW levels and liver-related disease progression and outcomes. One large sample size cohort study found that RDW is predictive marker for advanced fibrosis in patients with nonalcoholic fatty liver disease^26^. Fan et al. found that RDW is elevated in patients with CHB and that there is a positive correlation between these changes and CHB severity^27^. RDW is significantly increased in patients with HCC; it represents an easily measured marker of prognosis in these patients^28,29^.

Abnormalities including inflammation, erythrocyte fragmentation, oxidative stress, poor nutritional condition, and abnormality of erythropoietin function can cause significant variation in RDW^5,30–34^. Because these disorders and anemia correlate with liver disease severity, elevated RDW values might also be associated with the severity of liver disease. Inflammation results in impairment of erythrocyte maturation and entry of immature erythrocytes into the systemic circulation that results in elevated RDW values^19,35^. Study results suggest that inflammatory cytokines (e.g., tumor necrosis factor-α, interleukin (IL)-1β, and IL-6) inhibit iron metabolism and erythropoietin production. This process results in disorders of RBC synthesis and abnormal erythropoietin production^35–37^. Impairment of the balance between oxidant and antioxidant defenses are characteristic of the oxidative stress that occurs in liver disease. Erythrocyte homeostasis and survival is strongly affected by oxidative stress, and low serum antioxidant concentrations are correlated with increased RDW levels^38,39^. Taken together, this suggests oxidative stress results in increased RDW levels in patients with liver disease. A liver disease-associated poor nutritional status (e.g., iron deficiency, folate deficiency, vitamin B12 deficiency) can also result in abnormal RBC production and increased RDW levels^40^. Patients with chronic liver disease can experience the common complication of portal hypertension. This condition can cause splenomegaly with an associated increase in the rate of RBC destruction and release of immature RBCs to the systemic circulation and consequent increase in RDW^38,39^.

Our study did not find relationships between RPR, RLR, and severe DILI. One explanation for this result is that the patients were not further analyzed (because of the sample size) to determine whether they had liver cirrhosis, which would have influenced PLT levels. Innate/adaptive immune responses have key roles in the biological mechanisms of DILI^41^ and could also could affect blood cell levels. Consistent with the results of other studies, we found associations between dyslipidemia, drinking, and the severity of DILI^2^. The detailed mechanisms have been described in our previous study^2,41^.

There are some limitations to our study. First, the study’s retrospective design might have caused selection bias that resulted in underestimated sensitivity and overestimated specificity values in the serum models^42^. Second, detailed information about lipid-lowering drugs and hypoglycemic agents was not available. More study is needed to understand associations between drugs used for metabolic disease and severe DILI. Third, the patients included in our study were not combined with other liver disease to tried to avoid factors which might influence RDW value, however we cannot avoid other system diseases because that might the reason patients use drugs. Fourth, because the study did not include a large sample size, subgroup analyses by acute and chronic DILI could not be performed. The number of cases was limited by the requirements to include patients with a diagnosis of DILI with liver biopsy and exclude patients without complete medical information.

In conclusion, risk for severe DILI was associated with drinking, dyslipidemia, higher TBA levels, and higher RDW. This study found that RDW and TBA levels may be valid for non-invasive, real-time monitoring of DILI severity in Chinese patients.

## Methods

### Patients

Data from 2097 patients who underwent standard laboratory tests and liver biopsies (The First Hospital of Jilin University, China) between January 2010 and December 2019 were assessed for inclusion in this retrospective study. Test and biopsy results indicated that 187 patients had a diagnosis of DILI. After excluding the data from 23 patients who had incomplete medical information, the data from 164 patients with complete medical histories and laboratory information were included in the analysis.

Subjects were excluded due to the following The exclusion criteria: adopted were (1) co-infection with human immunodeficiency virus or hepatitis B virus (HBV) or hepatitis C virus (HCV); (2) the evidence or history or other evidence of hepatitis (3) presence of other types of liver disease, such as (e.g., non-alcoholic fatty liver disease or, alcoholic liver disease, et al.

The Independent Institutional Review Board of The First Hospital of Jilin University approved the study protocol and the use of data from human subjects. All participants in the study signed informed consent.

### Liver biopsy

Each ultrasound-guided percutaneous liver biopsy was performed using the Menghini technique. All liver samples were preserved in phosphate-buffered formalin and then paraffin-embedded and sectioned and stained for histology. Liver activity and fibrosis were scored using the Metavir system^43^. We excluded tissue sections with fewer than three portal tracts (i.e., as poor quality). The pathologists were blinded to all clinical data. If their conclusions were different, the slides were examined by a another experienced hepatopathologist in the UK who was blinded to all data.

### Diagnosis of DILI

Dyslipidemia was defined using criteria from the 2015 Chinese Medical Association guidelines for DILI diagnosis and treatment^44^: All patients’ s RUCAM score more and equal to 6, and the presence of liver injury was finally diagnosed based on liver histology of percutaneous liver biopsies.

### Diagnosis of DILI severity

The 2015 Chinese Medical Association guidelines for DILI diagnosis and treatment were used to classify severity into five categories (level 0: exposure to drug, but no liver injury; level 1: mild, typically reversible elevations of serum enzyme activities only, total bilirubin (TBil) < 2.5 the upper limit of normal (ULN) value and international normalized ratio (INR) < 1.5; level 2: more extensive injury with early impairment of liver function indicated by increases in alanine aminotransferase (ALT) and/or alkaline phosphatase (ALP), TBil ≥ 2.5 ULN or INR ≥ 1.5; level 3: serious clinical illness in conjunction with obvious jaundice and disabling symptoms (TBil ≥ 5 ULN and/or INR ≥ 1.5); level 4: increases in ALT and/or ALP, TBil ≥ 10 ULN or TBil increases ≥ 17.1  μmol/L per day, INR ≥ 2.0 or prothrombin activity < 40% in addition to secondary loss of other organ functions (e.g., encephalopathy or hepatorenal syndrome); level 5: liver required transplant or death of patient^44^.

### Diagnosis of dyslipidemia

Dyslipidemia was defined using criteria from the National Cholesterol Education Program Adult Treatment Panel III (ATPIII), which were total cholesterol greater than 240 mg/dL, HDL cholesterol less than 40 mg/dL, LDL cholesterol greater than or equal to 160 mg/dL, or triglycerides greater than or equal to 200 mg/dL^45^.

### RDW-to-lymphocyte ratio, RDW-to-platelet count ratio, and aspartate aminotransferase-to-alanine aminotransferase ratio

RLR was calculated using RLR = RDW (%)/lymphocyte (10^9^/L), RPR was calculated using RPR = RDW (%)/PLT (10^9^/L), and AAR was calculated using AAR = AST (IU/L)/ALT (IU/L).

### Study variables

Demographic characteristics (e.g., age, sex, drinking, smoking) and variables associated with the clinical presentation (presence of hypertension, history of hypersensitivity, presence of dyslipidemia, type 2 diabetes) were included in this study.

At the time of liver biopsy, fasting blood samples were obtained for standard laboratory tests. Retrospective data on white blood cell (WBC), neutrophil, and lymphocyte counts, and on hemoglobin, RDW, platelet counts, mean corpuscular volume, serum aspartate aminotransferase (AST), ALT, gamma glutamyl transferase, ALP, TBil, TBA, and globulin levels were obtained from the patient’s medical record.

The INR, prothrombin activity, and prothrombin time were also analyzed in the patients with DILI and were used to classify severity of liver injury.

### Statistical analysis

The results for continuous variables were calculated as median, and 25th and 75th percentile values and analyzed using two-tailed independent sample t-tests. Categorical variables were summarized as numbers/percentages and analyzed using Chi-squared tests. To adjust for potential confounding effects, multivariate logistic regression analyses were performed, including adjusted odds ratios (AORs) and 95% confidence intervals (CIs). We used SPSS software (version 13, SPSS Inc., Chicago, IL, USA) to perform the statistical analyses. All tests were two-tailed. We considered *P* values < 0.05 to be statistically significant.

Receiver operating characteristic (ROC) curves and the area under the ROC (AUROC) curve were used to evaluate and compare the accuracy of AAR, RPR, RLR, TBA levels, and RDW for the diagnosis of DILI severity. ROC curve analysis and Z-tests were used to compute and compare AUROCs, respectively (MedCalc Software bvba, version 16, Ostend, Belgium). Maximizing the sum of sensitivity and specificity or optimizing a specificity of at least 95% were used to obtain cut-offs. All methods were carried out in accordance with relevant guidelines and regulations.
